# Mechanical performance of co-deposited immiscible Cu–Ta thin films

**DOI:** 10.1038/s41598-020-74903-2

**Published:** 2020-10-20

**Authors:** Evan Raeker, Max Powers, Amit Misra

**Affiliations:** Department of Material Science and Engineering, University of Michigan, Ann Arbor, USA

**Keywords:** Structural properties, Synthesis and processing, Mechanical properties, Metals and alloys

## Abstract

The immiscible alloy Cu–Ta has the potential for enhanced mechanical performance in applications as a functional coating. To establish baseline mechanical properties, four Cu–Ta films were co-sputtered at the temperatures 23, 400, 600, and 800 °C and tested with nanoindentation at strain rates 5 $\times$ 10^−3^ s^−1^ to 10 s^−1^. Each film had a unique microstructure morphology. The hardness and elastic modulus of the four films were insensitive to strain rate changes. Instead, the measured properties were spatially dependent, particularly in the 600 and 800 °C films. In those two films, there is a bimodal deformation behavior due to Cu-agglomeration under protruding grains and planar Ta-rich regions. Increasing the indentation depth revealed shear band suppression which is related to a homogenous distribution of flow stresses for all four microstructure morphologies. Finally, the Cu–Ta hardness appeared to follow a rule-of-mixtures when compared to extrapolated data of Cu and Ta monolithic films.

## Introduction

Immiscible alloy films may have interesting mechanical properties due to the distribution of atomically sharp interfaces between the phase separated regions. The length scales of the phase separated regions and the character of the interfaces will influence key deformation mechanisms such as dislocation trapping and suppression of shear localization^1^ and may give immiscible alloys, sometimes in the form of metallic nanocomposites, a variety of properties including high yield strength, ductility, and elevated fatigue and wear performance. Copper-based immiscible alloys are capable of enhanced hardness and toughness even at elevated temperatures^2,3^. The thermally stable microstructure paired with heightened mechanical properties as a thin film^4^ make Cu–Ta a promising material for protective coating applications. The immiscibility of Cu and Ta enables a wide compositional range for the constituent elements while avoiding the formation of intermetallics which may severely embrittle the alloy.

Prior research on Cu and Ta monolithic films reveal that Cu’s mechanical performance is strain-rate sensitive^5^ and is generally ductile^6^ depending on the grain size. Nanocrystalline Ta films have hardness far exceeding bulk hardness^7^ and reduced or even negative strain-rate sensitivity depending on the Ta phase^8^. Combining Cu and Ta as an immiscible alloy increases its mechanical performance beyond what is possible with either solitary element. Multilayer Cu–Ta films have shown enhanced mechanical properties contingent on layer thickness but suffer from shear banding at nanometer-scale layer thicknesses^9^. The penchant for shear banding is attributed to flow localization at the interface which is a function of interfacial nature. Simulations on the similar Cu–Nb system show that dislocation motion, pile-up, flow localization, and strain hardening of multilayers highly depends on interface coherency and orientation^10,11^.

Moving beyond the multilayer design, co-sputtering of Cu and Ta film enables the access of three-dimensional nanoscale morphologies that can have provide high hardness while suppressing shear bands. Variable deposition rates and temperatures can produce a spectrum of morphologies as the Cu–Ta self-segregate into phase separated regions. The scale and orientation of the phase separated regions is important to limit shear banding, inhibit plastic flow localization, and induce interfacial-dominant deformation mechanisms to yield enhanced mechanical performance of these thin films. This is particularly true if there is nanoscale spacing between microstructural heterogeneities which may behave as barriers to dislocation glide. On such scale, Hall–Petch strengthening^12,13^ and confined layer slip will have significant influence on material strength.

**Figure 1:**
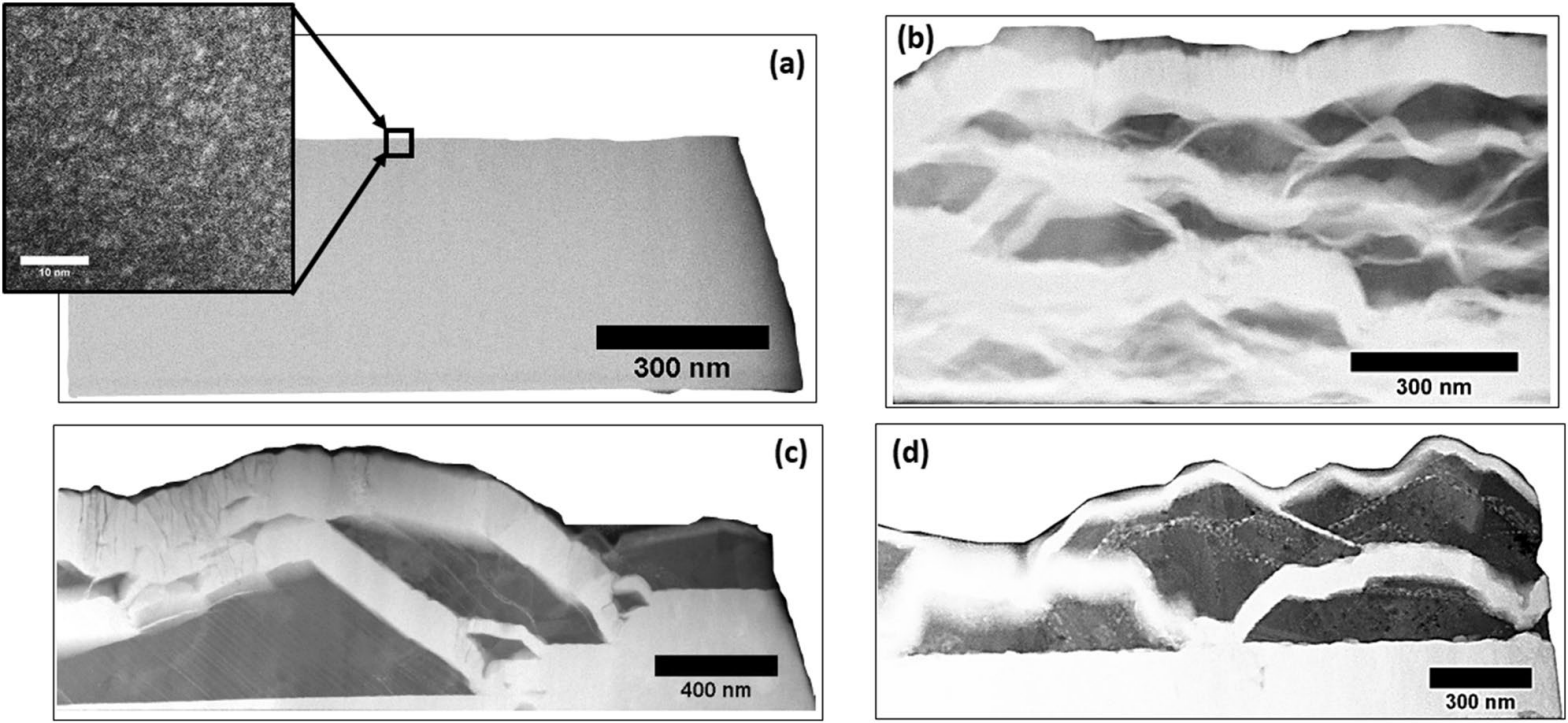
HAADF-STEM cross-sectional micrographs of the films of the four deposition temperatures. Ta-rich regions are lighter contrast, Cu-rich regions are darker contrast. (top left, (**a**)) 23 °C, with nanocrystalline Cu–Ta phase separated regions as indicated by high resolution TEM inset. (top right, (**b**)) 400 °C, alternating concentration modulations oriented perpendicular to the growth direction. (bottom left, (**c**)) 600 °C, Cu-rich agglomerates surrounded by Ta-rich veins, note the fine Ta-rich groups in the Cu-rich agglomerates. (bottom right, (**d**)) 800 °C, similar agglomerate-vein microstructure but with noticeably larger hierarchical features, particularly the Ta-rich clusters in the Cu agglomerates.

Previous works by this author^14,15^ have shown a direct relationship between co-deposited Cu–Ta film morphologies as a function of deposition temperature. The following morphologies were produced with the corresponding temperatures: 23 °C, nanocrystalline Cu and Ta; 400 °C, wavy-layered bicontinuous Cu–Ta oriented perpendicular to the film growth axis; 600 °C, agglomerated Cu surrounded by thin bands of Ta; and 800 °C, larger agglomerations of Cu with trace amounts of Ta trapped inside and Ta bands with trace amounts of Cu trapped inside. The 800 °C morphology is characterized as a hierarchical structure with features on multiple separate length scales as also observed in co-deposited Cu–Mo^16^. Figure 1 presents high angle annular darkfield (HAADF) scanning transmission electron microscopy (STEM) micrographs of cross-sectional samples to illustrate the four disparate film morphologies. Macroscopic deformation of these Cu–Ta films is required to gauge their efficacy in protective coating applications. The current work will characterize these four unique morphologies at low strain rates with high throughput testing via nanoindentation to build a base line for future experimentation at dynamic loading conditions. Of note is the effect of hierarchical morphologies on overall film mechanics. A metallic multilayered Cr/Cu–Cr displayed improved nanoindentation hardness due to the Cu precipitates in the Cu–Cr layer^17^. The hierarchical morphologies of Cu–Ta may replicate that behavior with precipitate hardening caused by the minority Cu and Ta phases.

An important consideration for PVD metallic films is the residual stress inherent to the non-equilibrium film deposition process. The heating and cooling during deposition will induce internal stresses caused by the coefficient of thermal expansion mismatch of the substrate and the metallic film. Sample calculation using the formula for biaxial strain and the coefficient of thermal expansion for Cu of 16 $\times {10}^{-6}$, Ta of 6.5 $\times {10}^{-6}$, and Si of 2.6 $\times {10}^{-6}$, all in units of m (mK)^−1^, and known elastic constants, yields a calculated stress of 60 MPa in a 50–50 at% Cu–Ta film deposited at 800 °C^18^. The calculated stress will be compressive upon heating during deposition and tensile upon cooling to room temperature. Compared to the measured hardness (and flow strengths inferred from the hardness) of the Cu–Ta films reported in this article, these residual stresses are very low.

In this investigation, the indentation response of Cu–Ta co-sputtered films at quasi-static strain rates is correlated with film heterogeneities and microstructure morphology. However, surface features such as faceted regions or protruding grains will alter the contact area of the arriving tip and can significantly influence nanoindentation measurements^19^. Thus, both the microstructure and surface topology will be considered when analyzing variations in measured indentation behavior.

## Experimental methods

### Thin film fabrication

The co-deposition used 3-in diameter Si substrates with a 1000 nm thick SiO_2_ surface layer. The substrates were ultrasonically cleaned in acetone, rinsed with isopropanol alcohol, then dried with compressed air. The PVD chamber was a Kurt Lesker PVD 75. The chamber maintained a base pressure below 6 $\times$ 10^−7^ Pa and the substrates were cleaned with an RF bias of 50 W for 120 s before deposition. The Cu–Ta films were co-deposited via the PVD process of DC magnetron sputtering using 2-in disk Cu and Ta targets with nominal purities of 99.999% for Cu and 99.95% for Ta. The Cu–Ta targets were simultaneously activated to co-deposit films with 50 at% Cu–Ta composition with a target throw distance of 5 in. Four separate depositions were performed at four distinct deposition temperatures: 23 (room temperature), 400, 600, and 800 °C. The deposition temperature refers to the temperature of the substrate that is heated via a heating coil to a consistent temperature during the respective deposition. All films were deposited to a nominal 700 nm thickness. After deposition, the samples were cooled to room temperature over a few hours in the high vacuum of the deposition chamber to prevent oxide formation on the film surface.

### Nanoindentation and imaging (SEM)

Nanomechanical properties including strain rate sensitivity and surface feature effects were tested using a standard Berkovich probe on a Hysitron 950 Triboindenter. Strain rate sensitivity was assessed over a strain rate range of 5 $\times$ 10^−3^ s^−1^ to 10 s^−1^. The indentation depth for the strain rate testing was 100 nm except when probing the flat, Ta-rich regions in the 600 °C and 800 °C films at an indentation depth of 80 nm. Indentations for strain rate sensitivity testing were spaced > 10 microns from each other and were organized in grids.

The piezo scanning functionality on the Triboindenter was used to generate topographical maps and target particular surface features for indentation. Higher resolution scans performed with a tip velocity of 1 $\times$ 10^−1^ s^−1^ were used to image the residual indent and confirm its location. Further imaging of the residual indents was performed with a Tescan Mira3 FEG scanning electron microscope. Images were taken in both secondary electron (SE) and backscatter electron (BSE) imaging modes.

## Results

### Strain rate sensitivity

The four deposition temperatures of 23, 400, 600, and 800 °C yielded four distinct microstructure morphologies as shown in Fig. 1. Expanding further on the feature length scale, the Cu and Ta nanocrystals in the 23 °C film are ~ 5 nm in diameter. At 400 °C, the Ta-rich and Cu-rich bands are 124 $\pm$ 5 nm and 83 $\pm$ 4 nm in width, respectively. For 600 and 800 °C, the Cu-rich agglomerates 30 to 500 nm in diameter with Ta rich veins 60 to 200 nm in width.

The film morphologies are a result of the complex relationship of the thermodynamic driving force for phase-separation, the kinetic energy of the landing adatoms, their corresponding surface interdiffusion length during deposition, and the residual stresses generated during elevated temperature co-sputtering. The initial understanding of immiscible alloy thin film growth is based on work by Atzmon^20^ and Adams^21^ which describe the behavior of the landing adatoms. The deposited two species land on the substrate surface and will preferentially phase separate, diffusing a specific interdiffusion length along the surface before being buried by the oncoming layer of deposition flux. The Atzmon model describes the surface interdiffusion length as:

1 $$\rho =\sqrt{\frac{{D}_{s}\delta }{\nu }}$$

where ${D}_{s}$ is the surface diffusivity of the species, $\delta$ is the thickness of the most recently deposited layer (on the order of interatomic spacing), and $\nu$ is the deposition flux. Typically, the adatoms will form locally de-mix to minimize system energy which influences the subsequent layers and as they grow into the homogeneous phase-separated regions. As the surface diffusivity is orders of magnitude higher than the bulk diffusivity and the films are deposited for a limited period, the bulk diffusion is considered negligible and surface diffusion is the primary facilitator of material flux during film growth. Atzmon and Adams determined that the microstructure morphology is contingent on the surface interdiffusion length of the landing species which is in turn controlled by the deposition rate, the deposition temperature, and the elemental selection.

The evolution of the Cu–Ta microstructures can be placed in the context of surface interdiffusion length during co-deposition. The films were deposited at four distinct temperatures (23, 400, 600, and 800 °C) with all other processing parameters held constant. At 23 °C, the landing Cu–Ta had limited kinetic energy and diffused a short distance before being buried by the oncoming flux, yielding the nanocrystalline Cu, Ta seen in Fig. 1a. Increasing the deposition temperature to 400 °C provides moderate kinetic energy to the species which enables phase separation into the wavy, lateral concentration modulation morphology of Fig. 1b. For 600 and 800 °C an interesting phenomenon is noted, the elevated temperatures resulted in preferential agglomeration of Cu surrounded by vein-like Ta. The rationale is the high surface interdiffusion length for Cu relative to Ta at such temperatures. The agglomeration occurs to lessen the interfacial energy by reducing the contacting area between Cu and Ta phase separated regions. The rapidity of the Cu agglomeration encapsulated trace amounts of the immobile Ta seen in Fig. 1c,d. A description of the surface morphology and phase distribution for each of the experimental Cu–Ta films can be found in a previous work by the authors^14^. Further microstructural characterization, including a discussion of the formation mechanisms are elaborated by the authors in an adjacent work^15^.

**Figure 2:**
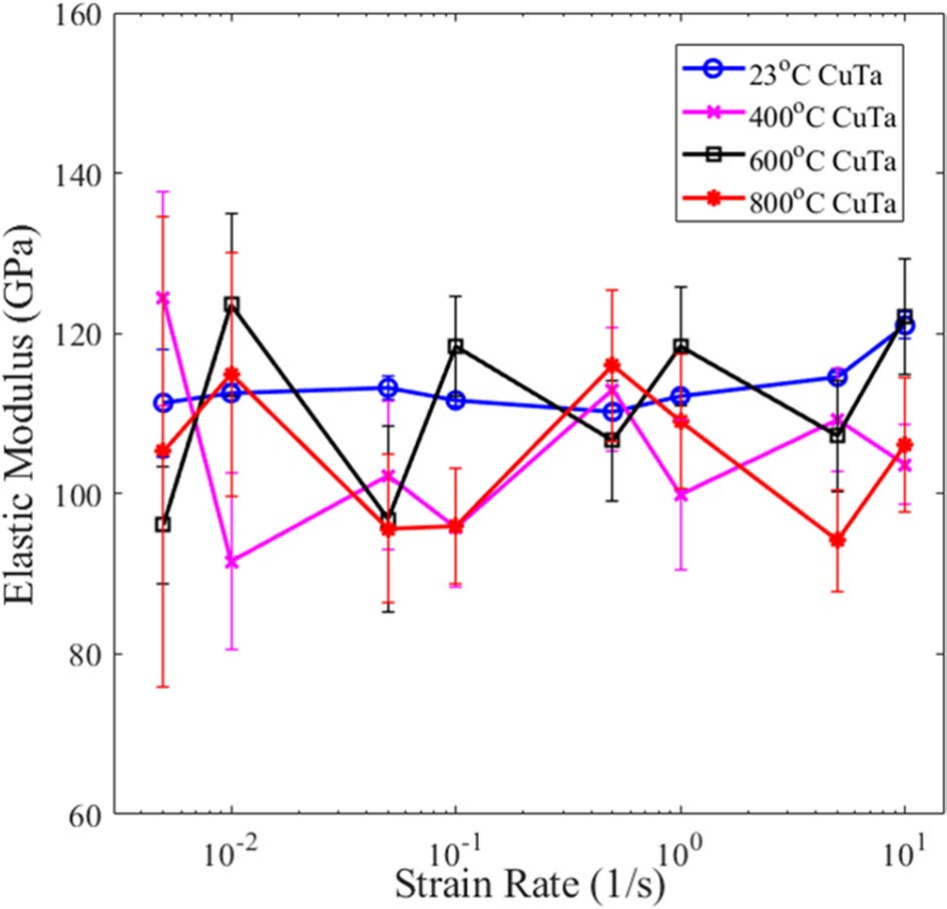
Average elastic moduli of Cu–Ta films deposited at varying temperatures tested across multiple strain rates. The error bars correspond to +/− one standard error.

**Figure 3:**
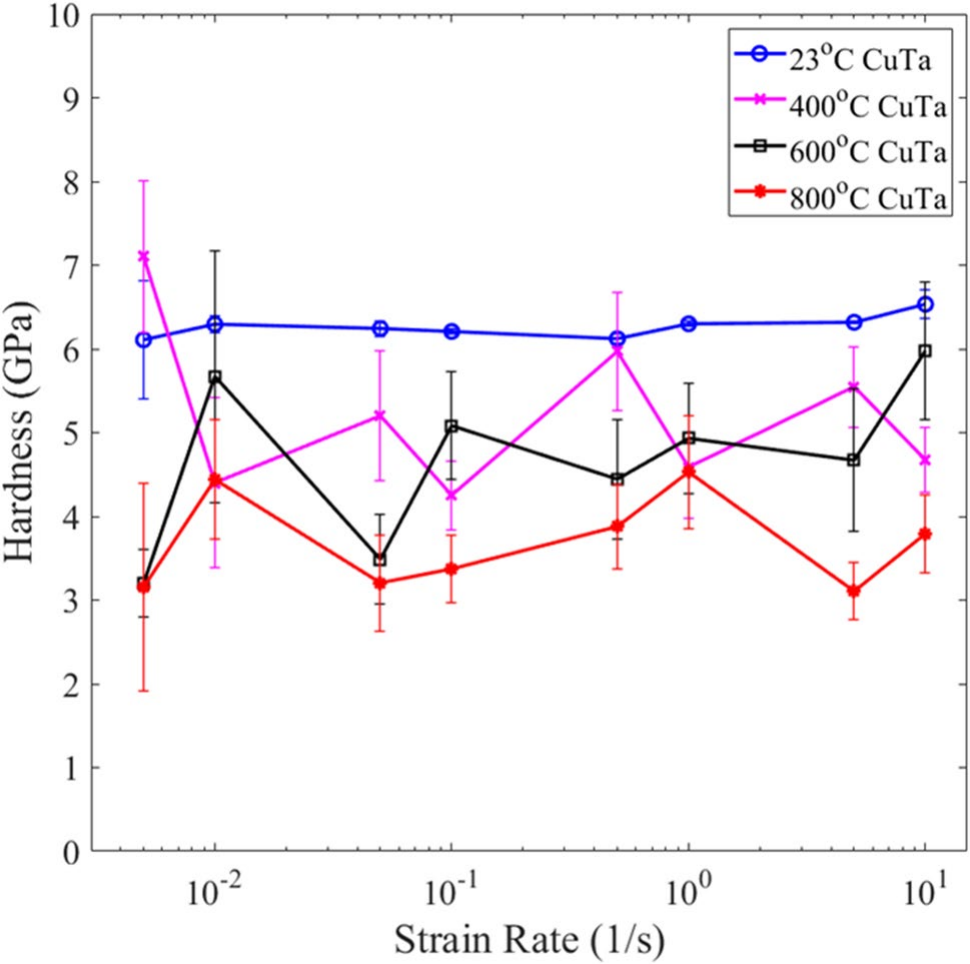
Average hardness of Cu-Ta films deposited at varying temperatures tested across multiple strain rates. The error bars correspond to +/− one standard error.

Nanoindentation performed at strain rates 5 $\times$ 10^–3^ s^−1^ to 10 s^−1^ indicated low strain rate sensitivity for all four Cu–Ta films as it pertains to microstructure. The strain rate sensitivity of the 23 °C film (*m*_Cu–Ta_) was estimated to be 0.005 by calculating the slope of the best fit line for the double log plot of flow stress and strain rate, where flow stress is approximated as $Hardness/3$. In comparison to its constituent elements, *m*_Cu–Ta_ is lower than that of pure Cu or Ta, with *m*_Cu_ ~ 0.1 and *m*_Ta_ ~ 0.05 for grain sizes around 10 nm^22,23^. We also find that *m*_Cu–Ta_ is lower than the strain rate sensitivity for multilayered Cu–Ta of similar composition. Multilayered Cu–Ta with equal layer heights was shown to have *m* ~ 0.05^24^. From Fig. 2, the average elastic modulus for each film has limited variance with respect to strain rate. This correlates with Fig. 3 as the films maintain relatively consistent hardness across the strain rates. Any differences across the Figs. 2 and 3 values are not a function of the bulk microstructure or strain rate, but rather the surface morphology. As will be elaborated later in section III b), the specific indentation location greatly alters the measured elastic properties.

In Fig. 2, all four films depicted average elastic moduli within the range of 105 GPa ± 15 GPa which is slightly below the elastic modulus of 121 GPa for monolithic nanocrystalline Cu films^6^. The experimental elastic modulus is also lower than the anticipated calculated rule-of-mixtures 50–50 at% Cu–Ta elastic modulus of 149 GPa^6,7,25^. The average hardness values in Fig. 3 ranged broadly at 4.5 ± 2.0 GPa. With a nanocrystalline Cu hardness of 1.89 GPa and a nanocrystalline Ta hardness of 11.6 GPa^6,7^, the experimental co-deposited Cu–Ta trended slightly under the calculated rule-of-mixtures 50–50 at% Cu–Ta of 6.75 GPa. A key observation from Fig. 3 is that the 23 °C film consistently performed on the higher end of the experimental hardness values. Averaging across all strain rates, the 23 °C film with nanometer-scale phase separated Cu–Ta displayed over 50% greater hardness than the other films.

### Spatially dependent mechanical performance

**Figure 4:**
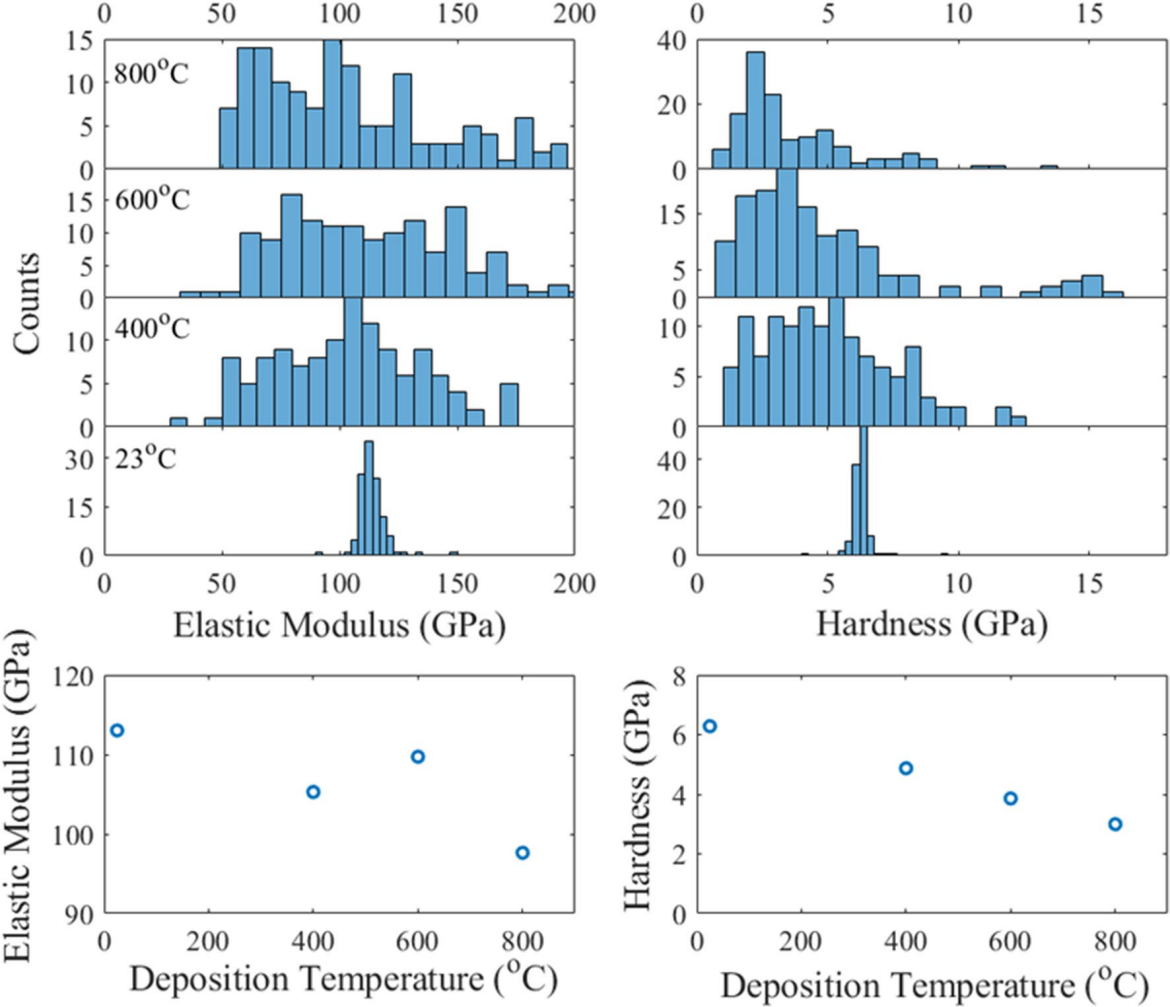
(top) Histograms representing the spatial distribution of mechanical properties across the film surface. The histograms are the combined data from the strain rate testing for each Cu–Ta film. (bottom) Median hardness and elastic modulus as a function of deposition temperature.

The substantial range in mechanical performance across indentations on a single film prompted investigation into factors other than microstructure that are influencing measured properties. The result is a high spatial dependence that is correlated to variant surface morphologies in the 400, 600, and 800 °C films. The histograms in Fig. 4 quantify the spatial dependence with respect to measured elastic modulus and hardness. The relative strain insensitivity in the high throughput testing suggests the indents from all tested strain rates can be used equivalently to compare the distribution of mechanical performance across the film surfaces. The histograms reveal a narrow normal distribution for both measured properties in the 23 °C film due to its planar, uniform surface of nanocrystalline Cu–Ta. The 600, and 800 °C films depict widened normal distributions for measured elastic modulus. The hardness histograms indicate bimodality with a majority cluster of low hardness indents followed by a minority cluster of elevated hardness values equivalent or exceeding the hardness of nanocrystalline Ta^25^. The 400 °C film has a wide distribution of elastic moduli but does not exhibit the hardness bimodality. An important metric of skewed distributions is the median value which is plotted against deposition temperature at the bottom of Fig. 4. In terms of general trends, elastic moduli and hardness both decline with respect to increasing deposition temperature.

Distinct surface morphology is the source of the spatially variant elastic moduli and bimodality of hardness in the 400, 600, and 800 °C films. The 400 °C film has coarse features with jutting grains (tens of nanometers-scale) that encompasses the whole surface. The 600 and 800 °C films both contain rough surfaces that can be classified into two cases. The first case is the coarsened, but uniform-in-height Ta-rich regions that also contain a minority phase of nanocrystalline Cu. The second case is a large protruding grain (hundreds of nanometers-scale) or “hillock” induced by stress relaxation and agglomeration of the more mobile species, Cu. The 600 and 800 °C films each have equal spatial fraction of the flat Ta-rich regions and protruding Cu-rich hillocks.

The wide normal distributions and bimodality encouraged examination of specific points on the film surface. In the 400 °C film, variations in mechanical properties can be attributed to the angle of contact between the Berkovich tip and the surface. Small grains jutting out of the surface and material displacement altered the contact area of the tip during the relatively shallow nanoindentation. The 600 and 800 °C films present a different case. A series of nanoindentations were performed that targeted specific flat regions between hillocks and sizable facets on the top of hillocks. A wide disparity in measured mechanical properties arose between the flat, Ta-rich regions and hillocks containing Cu-rich agglomerates.

**Figure 5:**
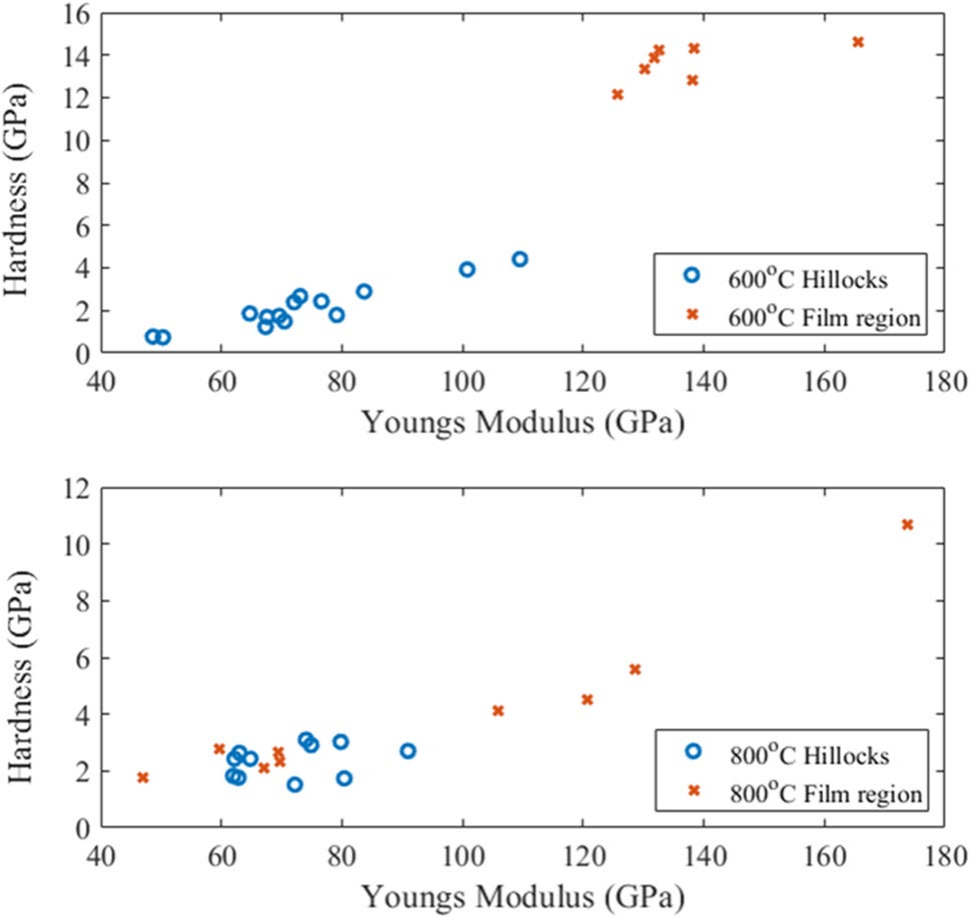
Elastic modulus and hardness of the two targeted surface morphology features. A hillock is a grain protruding from the film surface, in this case having a Cu-agglomerate core, and the film region denotes a planar Ta-rich surface. The error bars correspond to +/− one standard deviation.

The quantifiable data is represented in Fig. 5. As anticipated, the flat regions had mechanical properties akin to nanocrystalline Ta. The hillocks exhibited lower hardness and limited elastic deformation aligning with behavior observed for monolithic nanocrystalline Cu films. This was particularly interesting given that the hillocks tend to have thin layers of Ta-rich regions (~ 100 to 200 nm in width) that surround the Cu-rich agglomerates. The Ta-rich veins had limited effect as the agglomerated Cu underneath the protrusions readily displaced even under loads $\le$ 1000 μN. The bimodality in mechanical response was accentuated at 600 °C and less prominent at 800 °C which is directly correlated to the comparatively reduced spacing between Cu-rich agglomerates in the 800 °C microstructure. Spatial influence on properties will be an important consideration for any future testing at low or high strain rates on Cu–Ta films co-deposited at 600 or 800 °C.

**Figure 6:**
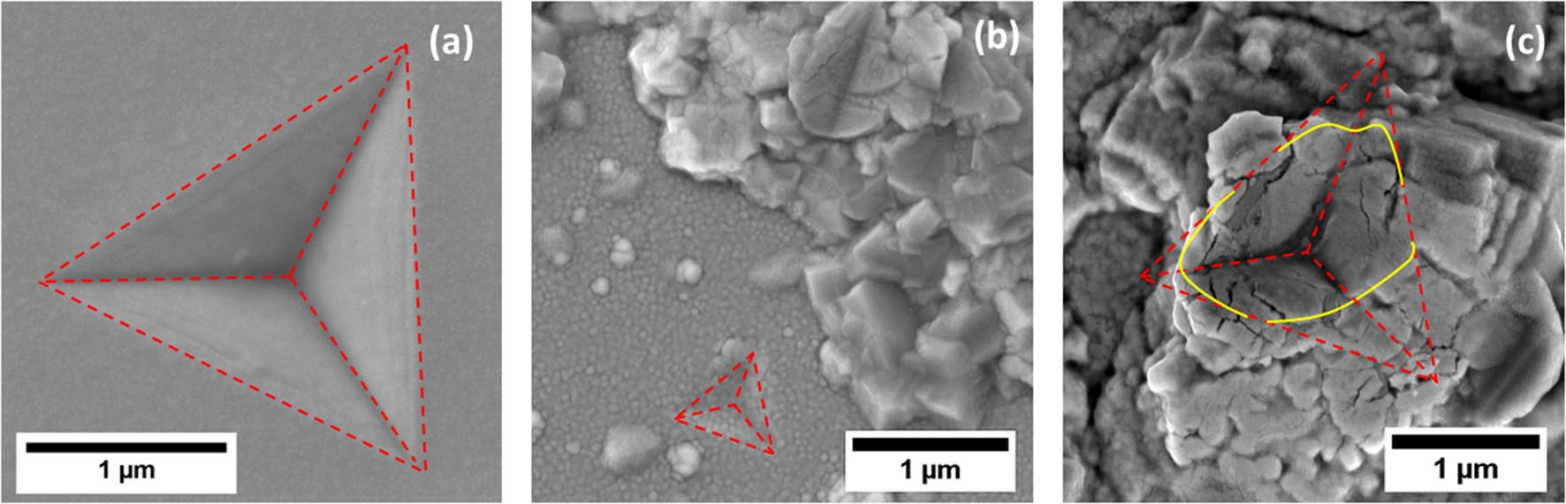
SE SEM images of residual high-load indentations on the surface of Cu–Ta deposited at 23 °C (**a**) and targeted surface morphologies for 600 °C (**b**) and 800 °C (**c**).

A series of load-controlled indents 200–300 nm in depth were performed to establish a qualitative understanding of the plastic deformation mechanisms in the surface features of the Cu–Ta films. These specific indents were not included in the data from the previously mentioned figures due to the increased strain of the substantial indentation depth and potential Si substrate effects. The SEM images of the indentation geometries is displayed in Fig. 6. In Fig. 6a, indentations on the 23 °C film maintain a uniform indentation geometry even at increased strain. This is a departure from previous nanoindentation research performed on Cu–Ta multilayers^9^. In the multilayer geometries, shear banding deformation was prevalent in samples with phase-separated Cu and Ta layers $\le$ 9 nm in width. In the current experiment, the phase separated Cu and Ta nanocrystals are ~ 5 nm in diameter but shear banding is absent from post-indentation SEM images. The lack of shear bands can be attributed to a homogenous stress distribution and limited regions for flow localization among the texture-less nanocrystals.

The SEM images in Fig. 6b,c exemplify the bimodality in the mechanical response of the 600 and 800 °C films. Both indents had the same applied force, but indentations on the film’s flat section in Fig. 6b showed marginal material displacement while Fig. 6c shows significant material displacement and a deeper indent into the hillock feature. Figure 6b also depicts the difficultly in isolating the measured properties from the Ta-rich regions as the indentation tip may contact small protruding grains on the film surface. Therefore, key source for error in this high throughput testing technique is varied tip contact area due to displaced material or non-uniform surfaces. The error is less applicable in the current experiment given the shallowness of the indents (80 to 100 nm) but can be sizeable in future research.

### Comparative results

To establish a comprehensive scope, the median values of hardness and elastic moduli in Fig. 3 of the 600 °C film are compared to nanoindentation data from Cu–Ta co-deposited at room temperature and annealed for a period of 2 h at 650 °C performed by Bahrami et al.^4^. Comparing films of nearly the same 50–50 at% Cu–Ta composition, the 600 °C film (current experiment) and as-deposited film have approximately equal hardness. While the annealed film is harder by ~ 2 GPa, the elastic moduli of the as-deposited and annealed films aligned with the calculated rule-of-mixtures 50–50 at% Cu–Ta elastic modulus of 149 GPa, showing higher stiffness than the 600 °C film.

**Figure 7:**
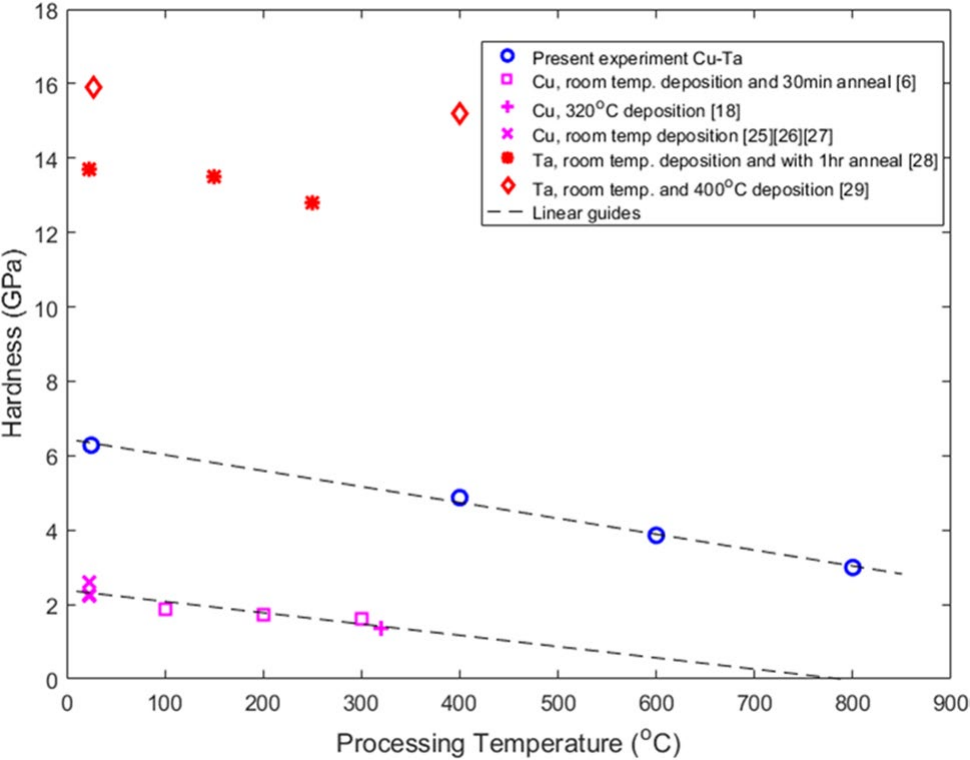
Hardness values as a function of temperature. Literature hardness values for monolithic Cu and Ta films annealed at various temperatures are compared to hardness values of Cu–Ta in the present experiment. The trend lines indicate general behavior and are not empirically-based.

One step further is to examine if the median hardness values in the current experiment continue to follow the rule-of-mixtures as a function of processing temperature. Figure 7^6,26–31^ compares the co-deposited Cu–Ta to literature monolithic Cu and Ta that have been annealed at various temperatures. The trend reveals the median hardness nearly follows the extrapolated average between the monolithic Cu and Ta at the same processing temperature. A lack of hardness data for the monolithic films processed at 600 and 800 °C leave ambiguity to the validity of the trend for the hierarchical structures in the 600 and 800 °C films.

## Discussion

All four heterogeneous Cu–Ta microstructures maintained minimal strain rate sensitivity for the range probed in this experiment, 5 $\times$ 10^–3^ s^−1^ to 10 s^−1^. Although the strain rate is varied across four orders of magnitude, the strain rates are still relatively low and would not activate high or very-high strain rate deformation mechanisms, ex. twinning or a shock response. Other reasons for the reduced strain rate sensitivity are the semi-coherent Cu–Ta interfaces with their discontinuous slip systems for dislocation transmission and precipitate hardening caused by Ta nanoprecipitates in the hierarchical morphologies. As both semi-coherent interfaces and precipitates impede the dislocation motion, any reduction of the activation barrier for dislocation motion caused by an increase in strain rate may be minimal which may be the source of the low observed strain rate sensitivity in Cu–Ta. Any conclusive statements on the exact influence of interfaces as impediments on dislocation motion in the material would have to be verified with in situ microscopy experiments in future studies. The same is true to validate any dislocation pile-up, bowing, or confined layer slip which may all be occurring at instances of sufficient strains in the material.

Nanoindentation is a high throughput method to determine the elastic properties of film surfaces. The key metrics of hardness and elastic modulus are extracted from the data on tip displacement as a function of applied load. Original nanoindentation studies conducted by Oliver and Pharr^32^ describe the elastic modulus as:

2 $$\frac{1}{{E}_{r}}=\frac{(1-{\nu }^{2})}{E}+\frac{(1-{\nu }_{i}^{2})}{{E}_{i}}$$

where ${E}_{r}$ is the reduced modulus which is calculated based on the slope of the unloading region of the indentation curve, $E$ and $\nu$ are the elastic modulus and Poisson’s ratio for the specimen and ${E}_{i}$ and ${\nu }_{i}$ are the same parameters for the indenter. The hardness is expressed as:

3 $$H= \frac{{P}_{max}}{A}$$

where ${P}_{max}$ is the peak indentation load, $A$ is the contact area of the Berkovich tip, and $H$ is the specimen hardness. Nanoindentation of the Cu–Ta provided two key sources of error that influenced the accuracy of Eqs. (2) and (3). Material displacement and jutting grains on the film’s surface affected both the contact area and the calculated reduced modulus. As a result, the measured hardness and elastic modulus could vary even when measuring film regions with nearly identical underlying morphologies. The error was less influential in the Cu–Ta deposited at 23 °C, moderate in the 400 °C sample, and especially prominent in the 600 and 800 °C films. The increased error was due to the ductile Cu-agglomerations under the hillocks which created large areas of uneven surfaces and experienced high material displacement even under reduced loads.

The authors sought theoretical and experimental methods to minimize the inaccuracy caused by material displacement and imprecise contact area. Formalism by Saha and Nix^33^ presented stiffness as a measurable property for mechanical performance. Normalizing the applied load by the square of the stiffness provides a metric that is insensitive to indentation depth and therefore projected contact area. However, the majority of literature on nanoindentation of Cu–Ta thin films provide results in the form of hardness and elastic modulus. To enable direct comparison to literature values, the authors chose not to adopt the metric proposed by Saha and Nix. Instead, the authors turned to experimental methods to reduce the error. With reduced applied load, shallow indentation depths, and precise location selection, it was possible to achieve consistent mechanical performance and avoid outliers in the data.

Given the shallowness of the indents and the emphasis on macroscopic properties, quantifiable properties were limited to the elastic regime. Mechanical performance as function of temperature for monolithic Cu or Ta films in Fig. 7 suggest that the Hall Petch relationship pertaining to grain size plays a key role in material performance. The specific Hall–Petch strengthening coefficient of ${k}_{h}$ = 5.2 $\times$ 10^–3^ MPa nm^1/2^ may explain the reduction in hardness with the coarser microstructures seen for Cu–Ta deposited at increased deposition temperatures^34^. However, further experimentation is necessary to draw conclusions on any Hall–Petch relationship or deviation thereof in these complex Cu–Ta films. The unique nature of co-deposited Cu–Ta microstructure would make grain size studies difficult, particularly in the hierarchical structures with the phase separated regions on multiple length scales. Additionally, the trapped Ta or Cu phases in the 600 and 800 °C films may be critical in preventing grain boundary rotation or grain boundary sliding but in situ measurements at higher strains would be needed to confirm this.

The absence of shear bands in the extended depth indentations of Fig. 6 suggest an equitable distribution of flow stress in all four films. This behavior contrasts with shear banding noted for Cu–Ta multilayers^9^ but aligns with homogenous stress distributions observed in Cu–Mo hierarchical structures^35^. The Cu–Mo hierarchical structures are akin to those seen in the 600 and 800 °C Cu–Ta with large agglomerations of Cu surrounded by a secondary structure. The hierarchical Cu–Mo reached compressive strengths upwards of 2.6 GPa at engineering strains exceeding 0.1 without shear band formation. The hierarchical structure suppressed shear banding due to uniform stress distributions throughout the Cu-Mo sample from dislocation pile-up and strain hardening effects paired with the variant spatial distribution of Cu–Mo interfaces which are weak in shear and susceptible to plastic flow localization. The same underlying factors can rationalize the suppression of shear banding of the current co-deposited Cu–Ta films.

The present work is distinguished from similar nanoindentation performed on co-deposited Cu–Ta because of the unique microstructures that arise from elevated deposition temperatures of nominally equi-atomic Cu–Ta films. With increased deposition temperatures, surface diffusion becomes the chief vehicle for material flux as landing adatoms will travel up to a specific interdiffusion length as determined by the kinetics of the adatoms, the driving forces for phase separation and chemical potential gradients induced by stress within the film, and the deposition rate of the oncoming layers. The dominance of surface diffusion coupled with high deposition temperatures yields the hierarchical structures with large agglomerates of Cu and the entrapment of the less mobile Ta and low mobility Ta impeding Cu movement. The frozen bulk approximation^20^ means little change in the film bulk during the deposition process. Co-sputtered Cu–Ta from literature^4,36,37^ deposited the films at room temperature and annealed the films for a period of time at elevated temperature. The annealing facilitates bulk diffusion and grain boundary movement. The resulting microstructures may be bicontinuous or concentration modulated but have not yet indicated any hierarchical formatting.

## Conclusions

The elastic macroscopic mechanical performance of co-deposited Cu–Ta films was tested using high-throughput nanoindentation to establish low strain rate baseline for eventual high-strain testing. The four Cu–Ta films deposited at 23, 400, 600, and 800 °C appeared to have little correlation between elastic modulus and strain rate as well as hardness and strain rate, and large variances were present in the measurements for the 400, 600, and 800 °C samples. The strain rate sensitivity of the 23 °C sample was calculated to be 0.005 for the probed strain rate range of 5 $\times$ 10^–3^ s^−1^ to 10 s^−1^. The large variances of mechanical performance at a given strain rate for the 400, 600, and 800 °C samples prompted further exploration into film surface morphologies. Indentations across the surfaces revealed a spatial dependence in the 600 and 800 °C films. The two films at elevated temperatures contained surface features identified as Ta-rich planar regions and protruding grains, hillocks, caused by Cu-agglomeration. Precise indentation into these two features showed a dichotomy of performance and revealed a source of error as indentation may potentially displace material and have incorrect contact area. Furthermore, the co-deposited Cu–Ta suppressed the formation of shear bands even as the indentation depth exceeded 10% of the film thickness. When comparing the median Cu–Ta film hardness to literature monolithic Cu and monolithic Ta as a function of processing temperatures, the hardness appears to follow a rule-of-mixtures for 50–50 at% Cu–Ta.
